# Supplementary material for: Primary break-up and atomization characteristics of a nasal spray
Source: PLoS One. 2020 Aug 5;15(8):e0236063. doi: 10.1371/journal.pone.0236063 (PMC7406034; doi:10.1371/journal.pone.0236063)
Supplement: Attachment — Submitted filename: Response to Reviewers.pdf [file pone.0236063.s001.pdf]

Response to Reviewer #1's Comments

ONE-D-20-09687

Primary break-up and atomization characteristics of a nasal spray

PLOS ONE

We thank reviewer 1 for a thorough review of the manuscript and thank them for their time in reading through the paper and providing valuable comments and suggestions to improve the quality of the paper. Below, we detail our responses, and corresponding revised sentences are colored in red.

Reviewer #1: The authors presented findings describing nasal spray atomization break up with close-up visualizations of the break up process. These results are important to advance current knowledge since they will help in developing realistic nasal spray models for topical nasal drug delivery. The manuscript was detailed and well written.

We appreciate the comments and encouraging words from Reviewer 1 which gives confidence to our team.

Response to Reviewer #2's Comments

ONE-D-20-09687

Primary break-up and atomization characteristics of a nasal spray

PLOS ONE

We thank reviewer 2 for a thorough review of the manuscript and thank them for their time in reading through the paper and providing valuable comments and suggestions to improve the quality of the paper. Below, we detail our responses, and corresponding revised sentences are colored in red.

1. The use of several advanced data treatments (detailed image analysis, Canny edge detection, a brief introduction using the Weber number to describe air-surface tension relationships) is included in the manuscript, but insufficient information regarding how these can be/will be used to parameterize the proposed LISA model is described. The reviewer is left to question whether a follow-up publication reporting on the development of the LISA model to describe these results is planned. The limited quantitative evaluation of the results or their application to a broader set of nasal sprays provided in the manuscript is a significant weakness.

Response: We agree that certain details were not provided in detail to highlight and showcase some of the innovations of the study - one that was missing is the technique of backlighting for shadowgraphy at high frame rates which has not been performed before. We also agree that the link between the measured data and LISA model was not as strong as could be, and that the details on how the results from this study apply to spray modelling have not been sufficiently provided in the manuscript. To improve this, we have added further discussion on the novel experimental technique and LISA model requirements. Its application towards the follow-up computational study of spray atomization using the LISA model is now discussed under a subsection "***Implementation in CFD modelling***" which relates the spray characteristics with quantitative data required in the LISA model and hollow cone injection approach of a nasal spray modelling.

Implementation in CFD modelling

Spray modelling has been well understood and validated for high-pressure applications such as industrial and combustion fuel sprays (De Villiers et al., 2004; Dos Santos et al., 2011). However, there are limited studies with accurate validation of low-pressure applications such as nasal sprays. The available primary break-up models, such as Huh and LISA (Huh, 1991; Senecal et al., 1999), were modelled for combustion sprays operating under high injection and combustion chamber pressure.

The current study aims to provide insight into the external and near-nozzle spray characteristics of a low-pressure nasal spray atomizing in atmospheric pressure. The high speed imaging was processed to obtain quantitative data to serve as a reference for initial conditions required for computational atomizer breakup models, such as the LISA model and an alternative approach involving explicitly defining the droplet

parcels at the disintegration/break-up length (liquid core length) based on our measurements from a nasal spray. Secondary break-up would be achieved through the Taylor-Analogy-Breakup (TAB) model which is suitable for low Weber number applications.

The LISA breakup model requires a spray cone and dispersion angle as inputs. The spray cone angle describes the spray plume development whereas the dispersion angle describes the liquid sheet fluctuation from the mean cone spray angle. The dispersion angle is an important parameter in the LISA break-up model, as it leads to the radial droplet dispersion from the mean cone spray angle. This parameter has not been reported for nasal spray applications and past studies have used a dispersion angle of 3° (Fung et al., 2012) by tuning the parameter to match a droplet size distribution. For engine sprays, the dispersion angle was generally set to 10° (Baumgarten, 2006; Suh et al., 1999). Our measured results showed an angle of 8.65° ± 0.64°.

An alternative to atomizer breakup models is to explicitly define the initial droplet conditions, which includes a choice of hollow-cone, ring cone and solid-cone, as well as a custom user-defined cone. The most likely choice of cone to best represent pressure-swirl atomizers would be the hollow cone model (Inthavong et al., 2012). In this approach the primary break-up (eg: LISA, Huh models) is not modelled, but rather the droplet conditions in the near-nozzle are defined. This information can be extracted from measurements, that includes droplet location at the break-up length where the liquid sheet disintegration occurs, and spray cone angle. The droplets are distributed on a hollow circular ring at a break-up length from the nozzle, and a droplet size distribution would be imposed based on an empirical Rosin Rammler distribution function within the nasal spray droplet size distribution range.

A fully-resolved model of spray atomization is computationally intensive and challenging. Our measurement data of near-nozzle characteristics includes spray cone angle, break-up length, ligament diameter (break-up diameter), and dispersion angle and aims to contribute to the existing dataset for spray atomization CFD model setup."

2. The authors conducted a somewhat limited study regarding spray formation using a single spray device containing a commercially-available formulation or water sprayed from the same device. The report would be strengthened by the inclusion of specific information about the spray actuator system used in the product tested (manufacturer, model, any performance or design specifications available) and about the properties of the specific formulation tested. The authors, instead, rely on the description of a range of

formulation variables obtained from the literature (Table 1). This results in the inability to build upon and potentially generalize beyond the specific results provided.

Response a): Information on the spray actuator system used in the product test has now been added in the "***Materials and methods***" section of the manuscript.

> *"A schematic of the experimental setup is shown in Fig 1a which contained an automated actuation system. The pneumatic actuator (model: SMC-CXSL10-10; ADI, Inc., Hatfield, Pennsylvania) was located under the bottle and was connected to a two-way solenoid valve, controlled by a programmable logic control (PLC) unit (model: Allen Bradley 1760-L12BWB). The spray bottle was fixed at its base onto the actuator, to avoid lateral motion during actuation. Speed controllers (model: SMC-AS2002F-06; Allied Electronics, Inc., Fort Worth, Texas) were mounted on the pressure lines to control the flow rate and thus maintain the speed of squeeze and release of the spray bottle. During actuation the spray bottle moved up and down with the platform it was attached to, while the spray nozzle position remained fixed. This allowed images to be captured with a fixed reference point. The strength of actuation force was controlled by a compressed air line and the pressure passing through was monitored and controlled by a pressure regulator to produce 5Bar (72.5Psi). The PLC unit consisted of mechanical switches, a timer and a counter which controlled the timing, and numbers of on and off activations of the solenoids. The mechanical signal emitted from the PLC unit was converted to a digital signal by a Schmitt trigger and was sent to trigger the digital camera for image acquisition".*

Response b): *An additional column is added in table 1 which shows the liquid properties of the drug formulation used in the current study and comparison made with water and the ranges of nasal sprays.*

Table 2 Liquid fluid properties of water compared with possible range of drug liquid properties and drug formulations

Fluid Properties at 25°C	Water	Nasal Sprays (range)	Nasal spray formulation used in current study[a]
Dynamic Viscosity (cP)	0.89	667-3761[b]	*923*
Surface tension (mN/m)	72	30-44	40.6

[a] properties of oxymetazoline HCl 0.05%

[b] (Doughty et al., 2010)) reported Afrin with 3761 cP, and Zycam with 655 cP

3. The authors provide results obtained at different fill levels in the spray bottle. The interpretation of these results would be enhanced if the length of the dip tube and the dimensions of the spray bottle were included or, preferably, if a relationship between the height of the fluid in the reservoir at each fill level relative to the depth of the dip tube in the fluid volume was provided.

Response: *The dimensions of the spray bottle and the length of the dip tube have now been added in the section "**Materials and Methods**" as:*

> *The measured length of the dip tube was 3 cm and the diameter of the bottle was 2.9 cm totaling the volume of the liquid to 20 mL in a bottle.*

In addition, volume of the liquid and corresponding length of the dip tube immersed in the liquid is provided in a new table (Table1).

Table 1 Measurements taken at different liquid volume of the bottle and corresponding length of the tube immersed representing full volume, mid-volume and low-volume bottle fills.

Bottle condition	***Volume of liquid (mL)***	***Corresponding length of the submerged tube (cm)***
Full-volume	*20-13.3*	*3-2*
Mid-volume	*13.3-6.7*	*2-1*
Low volume	*6.7-0*	*1-0*

4. Inadequate discussion of the effects of surface tension and viscosity are provided. Very general, qualitative descriptions of the effect of these variables (e.g. "…holds the fluid together and resists disruptions and instabilities…" (line 173-4)) are included while a mechanistic understanding of the effects of these variables are available in the literature and should be provided in summary form in the discussion in this manuscript.

Response: *We appreciate the reviewer's comments on liquid properties which are essential parameters that contribute to the characterization of spray atomization. A summary of the literature on the effect of liquid properties in primary atomization, spray plume patterns and characterization on secondary atomization have been added in the section "**Results and Discussion**".*

> *Nasal spray characteristics does not solely depend on nozzle design and internal nozzle flow. The liquid properties of the ejected fluid also influence spray characteristics. The addition of excipients such as buffers, solubilizers, preservatives, surfactants, bio-adhesive polymers and penetration enhancers lead to changes in the liquid properties of the drug formulation. Polymers such as methylcellulose (MC) and hydroxypropyl methylcellulose (HPMC) or polyacrylic acid derivatives act as bio-adhesives and enhance viscosity. Increase in liquid viscosity increases the break-up length as highly viscous fluid impedes liquid disintegration (Chung et al., 1998). This phenomenon was identified in the current study when the drug formulation, which was comparatively more viscous compared to water, had a somewhat longer break-up length (longer by ≈ 0.5mm).*

The formulation in the nasal spray used contains oxymetazoline hydrochloride 0.05% and exhibits thixotropic behaviour due to its viscosity-enhanced formulations. Its rheologic behaviour is demonstrated by its viscosity at resting state which is high (3761cP (Doughty et al., 2010)) but, under shear forces, its viscosity reduces, and hence the requirement that some nasal sprays must be shaken to reduce viscosity so the formula can easily pass through the atomizer. During spray atomization, the formulation stretches and swirls as it exits the nozzle orifice, thinning out and shearing into ligaments and later, into droplets. However, after depositing on the nasal mucosa, the formulation increases in viscosity in order to enhance its residence time on the surface and create the 'no drip' effect.

A lower surface tension liquid has higher tendency to disintegrate. The drug formulation has a lower surface tension than water. However, the effect of formulation on sprays can be described with the Ohnesorge number (Oh) which characterizes the effect of viscous to inertial and surface forces. Comparison of Oh numbers for the drug formulation and water indicate a greater influence of viscosity (Oh_{drug}=2-12Oh_{water}) with the assumption that inertial force is the same (ΔP=5bar actuation force). The liquid properties also influence secondary break-up as they affect the liquid core length and ligament diameter. An increase in formulation viscosity (with similar surface tension) is expected to reduce plume angle and produce both larger and more variable droplets irrespective of the nasal spray devices tested. However, studies have shown that plume angles were identical and had no influence on droplet size distribution when fluid with varying surface tension was used (with similar viscosity) and irrespective of the nasal devices tested (Dayal et al., 2004; Foo, 2007; Kooij et al., 2018).

5. The manuscript needs careful copy editing prior to the next submission. There are numerous typos, grammatical errors and generally careless mistakes in figure descriptors. The references are also poorly edited, especially with respect to consistency in journal names.

Response: *The manuscript has now been thoroughly revised. Each section of the manuscript has been significantly improved and the references are arranged consistently with respect to the journal names.*

References

Baumgarten, C. (2006). *Mixture formation in internal combustion engines*: Springer Science & Business Media.

Chung, I. P., & Presser, C. (1998). *Effect of Fluid Viscosity and Surface Tension on Liquid Sheet Disintegration of a Simplex Pressure-Swirl Atomizer.* Paper presented at the 11th Annual Conference on Liquid Atomization and Spray Systems.

Dayal, P., Shaik, M. S., & Singh, M. (2004). Evaluation of different parameters that affect droplet-size distribution from nasal sprays using the Malvern Spraytec. *Journal of Pharmaceutical Sciences, 93*(7), 1725-1742. Retrieved from http://dx.doi.org/10.1002/jps.20090

De Villiers, E., Gosman, A., & Weller, H. (2004). Large eddy simulation of primary diesel spray atomization. *SAE transactions*, 193-206.

Dos Santos, F., & Le Moyne, L. (2011). Spray atomization models in engine applications, from correlations to direct numerical simulations. *Oil & Gas Science and Technology–Revue d'IFP Energies nouvelles, 66*(5), 801-822.

Doughty, D. V., Diao, L., Hoag, S. W., & Dalby, R. N. (2010). Use of flexible weighted nasal spray dip tubes to improve product performance. *Journal of aerosol medicine and pulmonary drug delivery, 23*(2), 69-75.

Foo, M. Y. (2007). *Deposition pattern of nasal sprays in the human nasal airway—Interactions among formulation, device, anatomy and administration techniques*: The University of Iowa.

Fung, M. C., Inthavong, K., Yang, W., & Tu, J. (2012). CFD Modeling of Spray Atomization for a Nasal Spray Device. *Aerosol Science and Technology, 46*(11), 1219-1226. doi:10.1080/02786826.2012.704098

Huh, K. (1991). *A phenomenological model of diesel spray atomization.* Paper presented at the Proc. of The International Conf. on Multiphase Flows' 91-Tsukuba.

Inthavong, K., Yang, W., Fung, M. C., & Tu, J. Y. (2012). External and near-nozzle spray characteristics of a continuous spray atomized from a nasal spray device. *Aerosol Science and Technology, 46*, 165-177.

Kooij, S., Sijs, R., Denn, M. M., Villermaux, E., & Bonn, D. (2018). What determines the drop size in sprays? *Physical Review X, 8*(3), 031019.

Senecal, P. K., Schmidt, D. P., Nouar, I., Rutland, C. J., Reitz, R. D., & Corradini, M. L. (1999). Modeling high-speed viscous liquid sheet atomization. *International Journal of Multiphase Flow, 25*(6-7), 1073-1097. doi:10.1016/s0301-9322(99)00057-9

Suh, E. S., & Rutland, C. J. (1999). Numerical study of fuel/air mixture preparation in a GDI engine. *SAE transactions*, 2185-2201.
